# Possibilities of Fucoidan Utilization in the Development of Pharmaceutical Dosage Forms

**DOI:** 10.3390/md17080458

**Published:** 2019-08-05

**Authors:** Aleksandra Citkowska, Marta Szekalska, Katarzyna Winnicka

**Affiliations:** Department of Pharmaceutical Technology, Medical University of Białystok, Mickiewicza 2c, 15-222 Białystok, Poland

**Keywords:** polysaccharide, marine-derived, fucoidan, pharmaceutical formulations, multifunctional polymer, fucospheres

## Abstract

Fucoidan is a polysaccharide built from L-fucose molecules. The main source of this polysaccharide is the extracellular matrix of brown seaweed (*Phaeophyta*), but it can be also isolated from invertebrates such as sea urchins (*Echinoidea*) and sea cucumbers (*Holothuroidea*). Interest in fucoidan is related to its broad biological activity, including possible antioxidant, anti-inflammatory, antifungal, antiviral or antithrombotic effects. The potential application of fucoidan in the pharmaceutical technology is also due to its ionic nature. The negative charge of the molecule results from the presence of sulfate residues in the C-2 and C-4 positions, occasionally in C-3, allowing the formation of complexes with other oppositely charged molecules. Fucoidan is non-toxic, biodegradable and biocompatible compound approved by Food and Drug Administration (FDA) as Generally Recognized As Safe (GRAS) category as food ingredient. Fucoidan plays an important role in the pharmaceutical technology, so in this work aspects concerning its pharmaceutical characteristics and designing of various dosage forms (nanoparticles, liposomes, microparticles, and semisolid formulations) with fucoidan itself and with its combinations with other polymers or components that give a positive charge were reviewed. Advantages and limitations of fucoidan utilization in the pharmaceutical technology were also discussed.

## 1. Introduction

**Figure 1:**
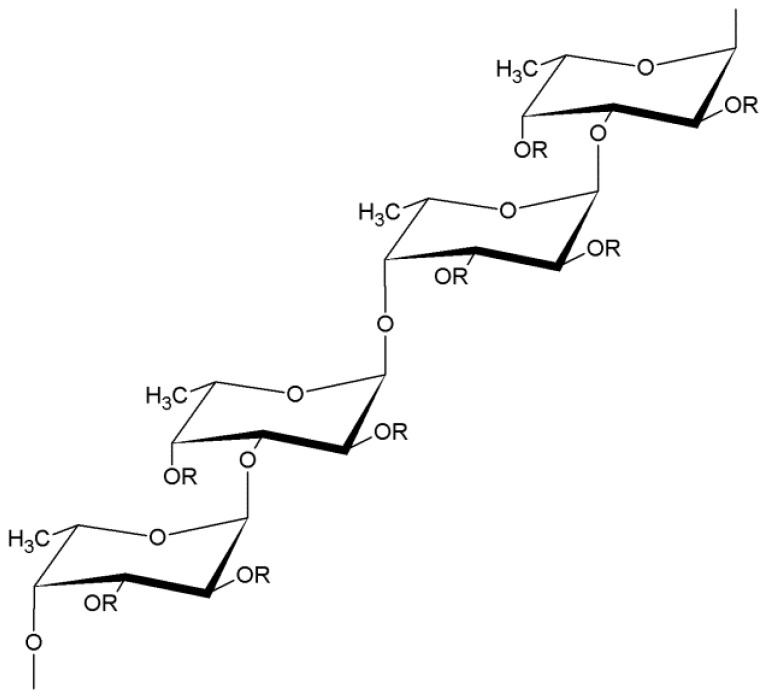
Scheme of the fucoidan structure; R- carbohydrate substituents: xylose, arabinose, rhamnose, glucose, galactose, or uronic acid and non-carbohydrate substituents: sulfate or acetate residues.

Fucoidan belongs to the large group of sulfated, rich in l-fucose polysaccharides, which was first found by Kylin in 1913 [1]. The main source of fucoidan are marine brown algae (*Phaeophyta: Laminariaceae, Fucaceae, Chordariaceae, Alariaceae*), but it has been also isolated from marine invertebrates such as sea cucumber (*Holothuroidea: Stichopodidae, Holothuriidae*), from sea urchins eggs (*Echinoidea: Strongylocentrotidae, Arbaciidae*) [2], and from seagrasses (*Cymodoceaceae*) [3]. The chemical structure and molecular weight of fucoidan are various in dependence of the species from which it is extracted, growth environment, season of harvesting and the method of extraction used [2,3]. Fucoidans isolated from the majority of brown algae are branched and have a backbone of alternating (1→3)- and (1→4)-linked α-l-fucose residues. Sometimes fucose molecules are connected by 1→2 linkage (Figure 1) [3,4]. Studies demonstrated that the most of echinoderms and some families of brown algae (*Laminariaceae* and *Chordariaceae*) are the source of fucoidan with linear chain built of (1→3)-linked α-l-fucose residues [3,4,5]. The negative charge of this biopolymer results from the presence of sulfate groups, which are mainly substituted on C-2 and C-4 and occasionally on C-3 positions [2,4]. Besides fucose, fucoidan may contain other sugars, for example: xylose, arabinose, rhamnose, glucose, galactose and uronic acid or even protein and acetyl groups. Both molecular weight (in the range between 10 and 2000 kDa), as well as the varied percentage shared in sugar and non-sugar components makes the analysis of fucoidan structure difficult [3,4]. Furthermore, the structure of fucoidan depends on the species of algae, but it can be different even for the same species thus the term “fucoidan” does not refer to one specific structure, but includes a diverse group of sulfated polysaccharides containing fucose.

The process of fucoidan extraction consists of several stages. The first step involves cleaning the algae, drying and grinding it or cutting fresh macroalgae into pieces and leaving in the dark, damp place to get exudate. Then different solvents (for example acetone, ethanol, mixture of acetone and ethanol or mixture of methanol, chloroform with water) are used to remove lipids, phenols and proteins. Fucoidan could be extracted by traditional solvent extraction (with water, ethanol or diluted acid) or using modern methods such as ultrasound-assisted extraction (UAE), microwave-assisted extraction (MAE) or enzyme-assisted extraction (EAE) [3,6]. New technique used to obtain fucoidan is also an extraction with 0.5% ethylenediaminetetraacetic acid (EDTA) at 70 °C. The unquestionable advantage of this procedure is that EDTA enables simultaneous removal of pigments and fucoidan extraction with high yield [7]. Regardless of the method used for extraction to obtain a specific polysaccharide, the next process is purification, most often by applying chromatographic methods or using molecular cut off membranes [6]. The wide interest of fucoidan in medicine and pharmacy is increasing due to its broad spectrum of biological activities. The bioactivity of fucoidan depends on several factors, predominantly on the content of sulfate groups and the molecular weight of the polysaccharide [3,4,8]. It was found that some fucoidans might exhibit antioxidant [9], anticoagulant [10], antibacterial [11,12], antifungal, antileishmanial [12], anti-inflammatory, and immunomodulatory [13] activity. Positive fucoidan impact on the treatment of patients with cancer diseases by improving the effectiveness of chemotherapy and reducing its side effects was also reported [2,3,14,15].

Despite many publications about fucoidan multidirectional biological activity, and many scientific reports on its use in drug delivery, there are still no registered drug products with fucoidan. It is currently available only in cosmetics, functional foods and dietary supplements as a regenerative, anti-inflammatory, and anti-cancer factor for patients with immune-compromised, musculoskeletal, cardiovascular, or gut diseases. However, fucoidan as synergistic anticancer agent has been subjected to clinical examination. It was observed that co-administration of fucoidan resulted in no significant impact on plasma concentrations of anti-cancer drugs, but might play a role in reducing side effects and in enhancing the therapeutic effects of conventional anti-cancer therapies [16,17]. The effect of oligo fucoidan in patients with non-small cell lung cancer treated with platinum-based chemotherapy has been tested in III-IV stage of clinical trials [18]. Fucoidan was also under clinical evaluation in patients with metastatic colorectal cancer treated with folinic acid, 5-fluorouracil, irinotecan, and bevacizumab. It was reported that fucoidan led to alleviate side effects of anti-cancer chemotherapy [19]. Fucoidan impact on the metabolism of fatty liver and liver fibrosis was also examined, but results have not been published yet [20].

## 2. Pharmaceutical Features of Fucoidan

Fucoidan is a pale brown or yellow powder with hygroscopic properties. It easily dissolves in water but is not soluble in organic solvents. Fucoidan solutions are not highly viscous, so it is not applied as thickening agent [21,22,23]. The viscosity of aqueous solutions of fucoidan is low and depends on many factors, including molecular weight, concentration, number of sulfate groups, degree of branching of the molecule, as well as temperature and pH [24].

In order to determine the basic properties of fucoidan obtained from *Laminaria japonica* (Weihai Century Biocom Seaweed Co., Ltd, Weihai, China), we examined its aqueous solutions (Table 1). The molecular weight of the tested fucoidan was 10.5 kDa. Both the content of fucose and sulphate groups were classified at a level higher than 27%. It was observed that with the increasing concentration of polysaccharide, the pH values of the solutions decreased (5.23 and 4.45 for 1% and 30% solution, respectively), while their viscosity was increasing and reached a value of 507 mPa·s for 30% solution, and no gelation was noted.

Similarly, Jae-Geun et al. have shown that water solutions of fucoidan from *Laminaria religiosa*, *Undaria pinnatifida*, *Hizikia fusiforme,* and *Sargassum fulvellum* possessed low apparent viscosity and were characterized by pseudoplastic behavior [21].

Viscosity of fucoidan is also influenced by algae species, presence of ions, or additional molecules. Comparing fucoidan solutions from *Saccharina longicruris*, *Ascophyllum nodosum*, and *Fucus vesiculosus*, the highest viscosity was observed when fucoidan isolated from *Fucus vesiculosus* was applied [25]. Viscosity of fucoidan solutions obtained from *Cladosiphom okamuranus* increased linearly with an increase of polymer concentration (up to 2%), and with the addition of sodium chloride, calcium chloride and sugar. It was stable at pH range from 5.8 to 9.5, indicating that fucoidan molecules are stable under acidic and alkaline conditions [22]. Contrary to other polysacharides, fucoidan does not show gelation ability [4,24,26], but upon fucoidan mixing with polymers with opposite net charge, based on electrostatic interactions, gels might be created.

It should be also emphasized that due to the ability to interact with growth factors, macrophages, cytokines and P-selectin, inhibition of the P-glycoprotein pump and α-glucosidase, as well as to open tight junctions in intestinal Caco-2 cell monolayer, fucoidan seems to be a valuable adjuvant in the pharmaceutical technology [2,3,4,5]. These properties allow not only to protect the drug substance, but also to strengthen drug activity, and they are described in more detail in further subsections devoted to specific formulations.

## 3. Toxicity of Fucoidan

Fucoidan utilizing in the pharmacy and biomedicine is possible because it is a non-toxic, biodegradable, and biocompatible compound [4,24]. Fucoidans from *Undaria pinnatifida* and *Fucus* vesiculosus were approved in the USA by Food and Drug Administration (FDA) as Generally Recognized As Safe (GRAS) category as food ingredients at levels up to 250 mg/day [27]. In Europe preparations containing *Fucus vesiculosus* are registered in Austria, Belgium, France, Poland, Spain, and the United Kingdom [28].

No changes occurred in rats receiving fucoidan from *Laminaria japonica* for 7 days, even at a dose of 4000 mg/kg body weight (b.w.). Subchronic toxicity studies conducted over 6 months have reported that no significant adverse effects were observed when fucoidan was orally administered to animals at the dose of 300 mg/kg b.w. per day, but when the dose was increased to 900 and 2500 mg/kg b.w. per day, clotting time significantly increased [29]. Nevertheless, low molecular weight fucoidan (LMWF) from *Laminaria japonica* did not lead to changes in b.w., water and food consumption, or hematological parameters after 28 days oral administration, even when the dose was 2000 mg/kg b.w. per day. Differences were observed only in two biochemical parameters—creatinine (CRE) and triglyceride (TG) levels were significantly lower than in rats receiving 0.9% saline solution. In histopathological examination of organs, including brain, heart, kidneys and liver, no discrepancies were observed between the control group and fucoidan treated rats [30]. However, when the source of fucoidan was *Cladosiphon okamuranus*, the dose that did not cause significant changes was 600 mg/kg b.w. per day and the anticoagulant activity of fucoidan was observed at higher doses (1200 mg/kg b.w. per day) [31]. No differences were observed in nutrition, b.w., hematological and biochemical parameters, as well as in organs subjected to necroscopy and microscopic examination in rats receiving up to 1000 mg/kg b.w. per day fucoidan from *Undaria pinnatifida*. Increasing the dose to 2000 mg/kg b.w. per day caused significant changes in mean corpuscular hemoglobin concentration (MCHC), alanine transaminase (ALT), TG, and high-density lipoprotein (HLD) levels [32]. Observed differences between the above-described studies indicate that the potential toxicity of fucoidans depends on the species from which it is obtained, as well as fucoidan molecular weight.

The safety of oral ingestion of fucoidan has also been studied in humans. Twenty healthy, adult volunteers received 4.05 g per day of mozuku fucoidan (isolated from *Cladosiphon okamuranus*) for two weeks. The blood samples showed a significant decrease in total cholesterol and low-density lipoprotein cholesterol. In turn, the concentration of chloride ions increased significantly. There were no changes in the parameters describing the liver and kidneys [33]. Similarly, it was shown in patients with osteoarthritis that application of fucoidan from *Fucus vesiculosus* at the dose 300 mg or from *Fucus vesiculosus*, *Macrocystis pyrifera,* and *Laminaria japonica* at 100 mg and 1000 mg did not affect the blood tests results or these changes were clinically negligible [34,35].

Safety of fucoidan was also evaluated at subcutaneous administration. Fucoidan from *Fucus vesiculosus* and *Laminaria japonica* (molecular weight 10–300 kDa) administered subcutaneously and orally did not cause negative changes in dogs with hemophilia A. Moreover, it also led to improvement of their hemostasis [36]. Similar conclusions about safety of fucoidan have been drawn from a study conducted on horses. The solution containing 2500 mg of fucoidan administered intraperitoneally during abdominal surgery did not have a negative impact on the results of laboratory tests. Most of the parameters did not differ between the test and the control groups, and the observed differences in the amount of leukocytes, neutrophils, antithrombin III, value of hematocrit, or prothrombin time were within the normal range [37].

Furthermore, the genotoxicity of high and low molecular weight fucoidan was also investigated with using both in vitro and in vivo model. In bacterial reverse mutation and chromosome aberration tests fucoidan from *Undaria pinnatifida* (regardless of the molecular weight) did not show toxic effect in concentrations 5000 µg/plate and 5000 µg/mL, respectively. Moreover, in micronucleus assay conducted in ICR mice, fucoidan at dosages up to 2000 mg/kg b.w. per day did not display evidence for genotoxicity [38,39].

## 4. Fucoidan Application in the Pharmaceutical Technology

**Figure 2:**
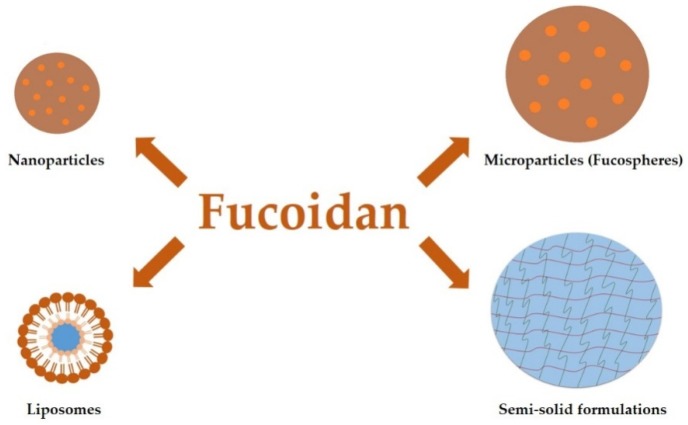
Schematic presentation of pharmaceutical dosage forms designed with fucoidan utilization.

Fucoidan plays an important role in the design of various dosage forms (Figure 2), especially nanoparticles and microparticles, films, or hydrogels [4,5,24]. The wide application of fucoidan in the pharmaceutical technology is due to its ionic nature. Negatively charged polysaccharide allows the formation of complexes with other, oppositely charged molecules. Polyelectrolyte complexation is the most commonly used technique for obtaining particles utilizing fucoidan. Other methods are coacervation, ionic cross-linking, self-assembly, and spray-drying [2]. Fucoidan-based particles served as carriers of various substances, including drugs (ciprofloxacin [40], gentamicin [41], doxorubicin [42], isoniazid and rifabutin [43]), growth factors (basic fibroblast growth factor—bFGF [44], proteins (bovine serum albumin [45], β-lactoglobulin [46]), and genes [47]. Interestingly, fucoidan is used not only as an excipient responsible for drug delivery, but also as a proper substance with therapeutic effects [12,48].

### 4.1. Nanoparticles

Nanoparticles are usually spherical particles with diameters from 10 to 1000 nm. Depending on the composition and the method of locating active substance in the nanoparticle, nanocapsules and nanospheres can be distinguished. In nanospheres the drug is homogenously dissolved or suspended in a polymer matrix. In turn, nanocapsules are the systems in which the drug is enclosed in a reservoir surrounded by a polymer membrane. Nanoparticles are valuable multicompartment carriers in modern pharmaceutical technology as they provide protection of the drug substance against chemical and enzymatic degradation, and enable controlled or targeted drug release. As a consequence, lower concentration of active substance is required to achieve a therapeutic effect, which contributes to the reduction of side effects [4]. Examples of fucoidan-based nanoparticles are presented in Table 2. To obtain nanoparticles with fucoidan utilization, self-assembly, and ionotropic cross-linking is commonly used. Self-assembly method is a process in which participating molecules, as a result of interactions between them, organize themselves into specific structures without introducing additional elements [49]. The ionotropic cross-linking is based on the interaction of the negatively charged fucoidan with the positively charged polymer molecule (e.g. chitosan) [50].

As shown in Table 2, various nanoparticles were tested to determine fucoidan potential in anticancer drug delivery. As model antitumor drugs, doxorubicin (DOX), copper sulfide (CuS), methotrexate (MTX), curcumin (CUR), silver nitrate (AgNO_3_), and cisplatin (CSN) were studied. In 2013, Lee et al. developed self-organized nanoparticles with acetylated fucoidan from *Fucus vesiculosus*. DOX was introduced into the nanoparticles through dialysis and it was released according to the first order kinetics. Fucoidan was tested as a drug carrier due to its immunomodulatory properties, ability to produce anticancer cytokines and drug efflux pump inhibition [51]. In turn, Lu et al. created nanoparticles by applying electrostatic interactions between the negatively charged fucoidan and the cationic peptide–protamine. The self-assembled complex was stable at pH 7.4 (corresponding to blood pH). When pH decreased to 4.5 (tumor cell pH) or 1.5 (stomach pH), electrostatic interactions became weaker, which led to increased diameter of nanoparticles and release of more than 90% of anticancer agent. The release of a smaller amount of DOX in the blood prevents side effects and increases its concentration in tumor cells. This pH-dependent release profile makes intravenous injection the best route for administration of these nanoparticles. Furthermore, using fucoidan that possesses the ability to interact with P-selectin present in the breast cancer cells (MDA-MB-231), resulted in the improved cell internalization and better inhibitory effect of designed nanoparticles than free DOX [52]. The polyelectrolyte complexing method (PEC) was used to combine fucoidan with polyethyleneimine (PEI). Similarly to the previously described study, when the medium pH was decreased, the increased release rate of DOX was noted. In vivo studies were performed using BALB/c mice with induced breast tumor. The results demonstrated that intravenously injected nanoparticles with DOX were characterized by stronger antiproliferative properties and higher tumor inhibitory activity than free DOX. The beneficial effects on the antitumor activity of the obtained nanoparticles were caused probably by immunomodulatory properties of fucoidan, which were confirmed in the in vitro studies [53]. In addition, fucoidan is characterized by the ability to improve the stability of metallic nanoparticles thereby contributing to the reduction of their toxicity. Fucoidan-coated gold nanoparticles with DOX caused a significant decrease in rabbit squamous carcinoma cells (VX2) viability. The most significant decrease in viability of the examined cells was observed after using both the discussed nanoparticles and laser irradiation. Similar conclusions were drawn from the in vivo studies conducted in rabbits with eye tumor. Only in the group treated with nanoparticles and laser, complete eradication of the tumor was observed after 6 days, and most importantly, there was no relapse after 14 days [42]. In turn, Jang et al. used the layer-by-layer technique for the synthesis of CuS nanoparticles alternately coated with fucoidan and polyallylamine hydrochloride (PAH). Based on the obtained results, it was found that the applied nanoparticles showed stronger anticancer effect than their components used separately. Obtained nanoparticles not only improved the intracellular transport of fucoidan, which possesses the ability to induce apoptosis, but it also provided favorable photothermal features. This was confirmed in the in vitro studies using human cervical cancer cells (HeLa) and human lung adenocarcinoma cells (A549), and in thein vivo studies in mice injected with these cells [54]. The same ingredients (fucoidan and PAH) were used to create nanoparticles with MTX via self-assembly method. MTX was released according to the first order kinetics and it showed stronger in vitro inhibition of HeLa and MCF-7 cell proliferation than unbounded drug. It should be noted that fucoidan and PAH complex was biocompatible and did not affect cells viability [55].

In nanoparticles the combination of fucoidan and chitosan or its derivatives is very commonly used, which is related to the properties of both polysaccharides. The positive charge of chitosan allows electrostatic interactions with sulfate groups of fucoidan, leading to the formation of complexes. In addition, chitosan is characterized by antifungal and antibacterial activity. Due to its ability to muco-adhesion and overcoming epithelial barriers, it facilitates the transport of active substances. Importantly, similar to fucoidan, it is biocompatible and its acquisition costs are relatively low [40,56,57]. In 2012, Silva et al. compared the activity of fucoidan–chitosan nanoparticles with the effect of fucoidan solution. Nanoparticles obtained by coacervation method were biocompatible with human epithelial cells (Caco-2) from colon adenocarcinoma and had the ability to open tight junctions between them. As a result, nanoparticles showed better permeability and stronger anticoagulant effect than the fucoidan solution per se [56]. Applying self-assembly method, Barbosa et al. produced nanoparticles and as the positive charge provider, chitosan was used. MTX loaded nanoparticles were nontoxic for both fibroblasts and keratinocytes when drug concentration did not exceed 50 µg/mL. The strongest anti-inflammatory activity manifested by the inhibition of Il1-β, Il-6, and TNF-α production and the greatest penetration of the active substance through the pig’s ear skin provided formulation with the highest fucoidan:chitosan ratio (5:1) [57]. Using the same electrostatic interactions between fucoidan and chitosan, Huang et al. created pH-sensitive nanoparticles for oral administration of CUR, which is characterized by limited bioavailability due to its poor solubility and sensitivity to environmental conditions of the gastrointestinal tract. Whereas at pH 1.2 CUR release was inhibited, at pH 7.0 significant increase in drug release was observed. Hence, the discussed nanoparticles can be considered as a carrier enabling oral administration of CUR, providing protection against degradation in the stomach and adequate bioavailability after absorption in the intestine [58]. Similar conclusions were also drawn from the studies in which CUR was placed in nanoparticles obtained from fucoidan and o-carboxymethylchitosan by ionic crosslinking. Controlled release of CUR in the gastrointestinal tract, its reduced cytotoxicity to mouse fibroblasts cells (L929), and increased cellular uptake by Caco-2 cells constitute the positive effects resulting from CUR encapsulation in nanoparticles [59]. By using thiolated fucoidan and arginine-modified chitosan it was also possible to obtain nanoparticles with dextran (DTX) or CUR. Due to the fact that utilized polysaccharides possess the ability to open tight junctions in intestinal Caco-2 cell monolayer, and thiolated fucoidan inhibits the activity of the P-glycoprotein pump, the penetration of both drugs were significantly better. Encapsulation of CUR as model hydrophobic compound into nanoparticles improved its water solubility and stability [60].

To deliver antibiotics to the lungs, fucoidan and chitosan composed nanoparticles via simple self-assembly method were designed. Antioxidant activity of the obtained nanoparticles (confirmed by the test of reactive oxygen species with lipopolysaccharides and by the method of scavenging 1,1-diphenyl-2-picrylhydrazyl radicals), stability in phosphate buffered saline, no negative effect on the viability of A549 cells at a concentration from 0.37 mg/mL to 3 mg/mL and controlled release of gentamicin make them an auspicious pulmonary drug delivery system [41]. Similar conclusions were drawn from the study in which nanoparticles with gentamycin were obtained by ionotropic crosslinking and applied intratracheally. The biphasic antibiotic release profile, as well as the antibacterial and antioxidant properties of fucoidan, resulted in stronger growth inhibition of *Klebsiella pneumoniae*, simultaneously limiting the potential nephrotoxic and ototoxic effects of gentamicin [50].

The protective properties of fucoidan were used to provide stability for silver nanoparticles. The fucoidan–chitosan complex coated silver nanoparticles, produced by self-assembly method, showed antibacterial activity both against Gram-positive (*Staphylococcus aureus*) and Gram-negative (*Escherichia coli*) bacteria. Moreover, the use of nanoparticles at a concentration of 50 µg/mL showed considerable cytotoxicity to HeLa cells [61]. In turn, Elbi et al. investigated the activity of ciprofloxacin loaded into chitosan nanoparticles obtained by ionic crosslinking using tripolyphosphate and subsequently coated with fucoidan. As fucoidan reacts with scavenger macrophages receptors, the obtained nanoparticles were characterized by better cellular uptake and thus led to more effective eradication of *Salmonella*. Research conducted on infected by *Salmonella Drosophila melanogaster* confirmed that ciprofloxacin (in the form of free drug, chitosan nanoparticles, or fucoidan coated chitosan nanoparticles) at concentrations four times higher than its minimum inhibitory concentration (MIC) did not affect the survival of the flies. However, the use of antibiotics at a concentration four times higher than minimum bactericidal concentration (MBC) caused the greatest survival rate of flies treated with the drug in the form of particles coated with fucoidan. Moreover, in this group the greatest decrease in bacterial load was noted, which confirmed the stronger antibacterial activity of ciprofloxacin enclosed in fucoidan coated chitosan nanoparticles, both in comparison to the free drug and to the form of chitosan nanoparticles [40]. By using the layer-by-layer method, Pinheiro et al. received nanoparticles made of alternating layers of chitosan and fucoidan deposited on polystyrene core. After removing the polystyrene element, nanoparticles were filled with poly-l-lysine (PLL), which is characterized by antibacterial properties. The satisfactory encapsulation efficiency (about 45%) obtained by the adsorption of PLL on the core before its removal, as well as the pH-dependent release of the active ingredient from the nanoparticles, make them a promising drug carrier [62].

The peptide therapy is becoming increasingly important in medicine and pharmacy. However, due to poor bioavailability, which results from peptide degradation in digestive tract and poor absorption, oral administration is limited. Tsai et al. have recently developed nanoparticles with trimethyl chitosan and fucoidan as carrier for insulin. Greater insulin protection against pH changes and better penetration across the intestinal epithelium was observed. It was also shown that fucoidan possesses the ability to inhibit α-glucosidase activity. Obtained nanoparticles at concentration of 2 mg/mL inhibited activity of this enzyme at ratio of 33.2% [63]. Additionally, the combination of fucoidan and chitosan provided protection for the basic fibroblast growth factor (bFGF). Nanoparticles were obtained by the ionotropic cross-linking. The effectiveness of encapsulation (EE), due to the ability of fucoidan to bind amino acids on the surface of bFGF, increased with increasing fucoidan content (for nanoparticles prepared from chitosan and fucoidan in a ratio of 10:1 and 1:10, EE was 80.9% and 90.4%, respectively). Moreover, the same relationship regarding the contribution of fucoidan was observed with respect to the protection of bFGF by enzymatic degradation and temperature inactivation. Nanoparticles at a concentration of 125 ng/mL showed no toxicity to pheochromocytoma PC12 cells, and due to the fucoidan and chitosan content therein, the amount of bFGF required to neurite extension was significantly lower than free bFGF [44].

It is worth noting that biological activity of nanoparticles built from fucoidan depends on the properties of this polysaccharide and the form in which it was used. In 2017, Juenet et al. obtained by redox radical emulsion polymerization (RREP) fucoidan-based nanoparticles. Due to the use of fucoidan, which has an affinity for P-selectin present on thrombocytes, the ability of nanoparticles to bind to the activated plates was greater. The fluorescence intensity observed after perfusion of both the fucoidan coated nanoparticles without recombinant tissue plasminogen activator (rt-PA) and with it was significantly higher than those in which no fucoidan was used. In addition, nanoparticles containing rt-PA and fucoidan showed the strongest thrombolytic activity in studies conducted in C57BL/6 mice. The thrombus density after their application decreased to about 30%, whereas after injecting nanoparticles without fucoidan, the value of this parameter was about 66%. It was found that nanoparticles not only protected rt-PA, but because of the ability of fucoidan to imitate P-selectin glycoprotein ligand-1, they improved its thrombolytic activity [64].

In turn, Lira et al. compared the properties of isobutylcyanoacrylate (IBCA) nanoparticles coated with fucoidan, which were obtained by two different methods. Anionic emulsion polymerization (AEP) allows the creation of stable formulations without the addition of DXT, whereas in the case of REEP only those containing up to 25% of fucoidan in the polysaccharide mixture were stable. Cytotoxicity was assessed after 48 h of nanoparticles incubation with macrophages J774 or fibroblasts NIH-3T3. The method of obtaining nanoparticles had a major impact on the cytotoxicity of nanoparticles only in relation to macrophages J774, because the IC50 value for nanoparticles formulated by AEP was 1.3 ± 0.2 μg/mL, and by REEP 9.6 ± 0.5 μg/mL. Interestingly, distribution of nanoparticles in these cells was dependent not on the method used for their production, but on fucoidan presence. Fucoidan did not affect the interaction of nanoparticles with fibroblast cells, but it also modulated nanoparticles uptake and distribution within macrophages [65]. The safety of using nanoparticles was assessed by Cai et al. Core of nanoparticles was made of poly(lactic-co-glycolic) acid (PLGA) and then it was covered with alternating layers of poly-L-ornithine (PLO) and fucoidan using the layer-by-layer (LbL) technique. Even at concentration of 100 µg/mL, they did not affect the morphology and proliferation ratio of breast epithelium (MCF-10A) cells. In addition, the hemolysis test on fresh blood from rabbits proved that by closing the PLGA in the capsule made of fucoidan and PLO, its biocompatibility increased (hemolysis rate for LbL and PLGA nanoparticles at concentration 100 µg/mL was 2.67% and 3.85%, respectively). Destructive effects on the liver, changes in nutrition and weight were not observed in mice after intraperitoneal injection of these nanoparticles at concentration of 10 mg/mL. The obtained results confirmed that such connection of fucoidan and PLO, not only protected active ingredient, but also improved its biocompatibility [66].

Interestingly, Hwang et al. prepared nanoparticles for colonic drug delivery by combining CSN and pure fucoidan. In order to obtain nanoparticles, two methods were used, differing in the order of dissolution of the components. Appropriate amounts of fucoidan were dissolved in 2 mg/mL CSN solution, or appropriate amounts of CSN were dissolved in 10 mg/mL fucoidan solution. Fucoidan nanoparticles tested as CSN carrier not only decreased toxicity of CSN against RAW264.7 macrophage cells, but they also improved its cytotoxicity in HCT-8 tumor cells [67].

Fucoidan was used to form shell of nanostructures dispersible in aqueous media. The source of polymer composed of β(1-3)-glucuronic acid substituted at the C4 position with fucose was the brown seaweed *Spatoglossum schroederi*. Chemical modification with hexadecylamine allowed to produce amphiphilic nanoparticles stable in water for up to 70 days. In order to determine the use of nanoparticles in the treatment of tumors, their effect on the proliferation and viability of various cell lines at 50, 100, and 500 µg/mL was evaluated by MTT test. It is based on the reduction of the water-soluble MTT dye, which is a triazole salt (3-(4,5-dimethylthiazole-2-yl)-2,5-diphenyltetrazolium bromide) to an insoluble dark blue formazan, the amount of which is proportional to the number of viable cells. This study showed that the produced nanoparticles did not affect Chinese hamster ovary (CHO) and mouse monocyte macrophage (RAW) cells. Antiproliferative activity was noted against rabbit aorta endothelial cells (RAEC) and all tested tumor cells: human renal cell carcinoma (786), human hepatocellular carcinoma cells (HepG2) and human marrow stromal cells (HS-5) [68].

### 4.2. Liposomes

Liposomes are specific type of structures, which size usually does not exceed 100 nm. The core formed by the aqueous environment is surrounded by the phospholipid bilayer. The main reason for the use of liposomes is the possibility of targeted therapy, which in addition to the improved effectiveness of the treatment, allows to reduce drug side effects [69]. The antitumor effect of LMWF encapsulated in liposomes was compared to the activity of fucoidan with molecular weight 2–10 kDa and 80 kDa using human osteosarcoma cells (143B). It was shown that apoptosis induction by activating caspase pathway was significantly stronger than by native fucoidan (95% and 25%, respectively). Similar conclusions were observed in studies in C3H mice that were previously injected with murine osteosarcoma cells (LM8). In the group receiving orally for 28 days liposomes with fucoidan or unprocessed fucoidan in the amount of 100 mg/kg per day, there was a significant decrease in both volume and weight of the tumor compared to mice receiving only water. However, better results were noted when fucoidan liposomes were applied. Furthermore, liposomes were characterized by better penetration through the Caco-2 cell layer. In turn, native fucoidan more strongly inhibited spontaneous metastases to the lungs. None of the forms (unprocessed fucoidan and fucoidan liposomes) led to negative changes in animal weight [70].

### 4.3. Microparticles

**Figure 3:**
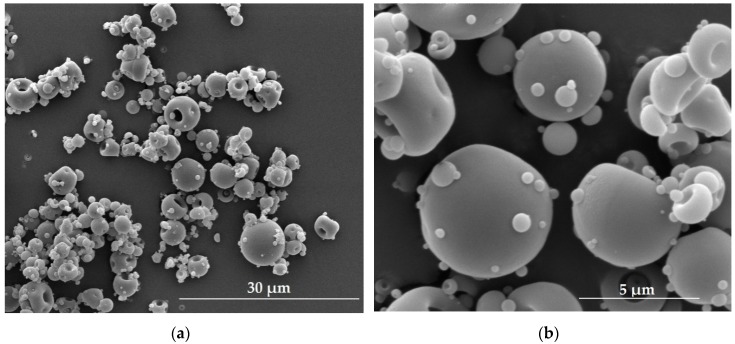
Scanning electron microscope (SEM) pictures of fucospheres: (**a**) magnification ×5000; (**b**) magnification ×20,000.

The term microparticles refers to particles with sizes ranging from 1 µm to 1000 µm. Analogously, as in the nanoparticles discussed above, among these group of particles microspheres and microcapsules can be distinguished. Microspheres prepared with fucoidan utilization are termed ‘fucospheres’. The morphology of fucospheres developed by our research team is shown in Figure 3. They were successfully obtained by the spray drying of 3% aqueous fucoidan solution (Table 1) using the Mini Spray Dryer B-290 (Büchi, Flawil, Switzerland) with experimental parameters set as follows: inlet temperature 122 °C, outlet temperature 62 °C, pressure 60 mmHg, aspirator blower capacity 100%, feed rate 2.1 mL/min.

According to the literature, preparation of fucoidan-based microparticles is usually performed with additional copolymers or positive charge donors (Table 3). In 2006, Sezer et al. proposed the use of fucospheres obtained by interaction between the negatively charged fucoidan and positively charged chitosan and, as a model protein, bovine serum albumin (BSA) was used. It was noted that with increasing fucoidan content in the fucospheres, BSA encapsulation capacity increased and the value of zeta potential decreased. Formulated microspheres provided three-phasic BSA release profile [45]. Interactions between fucoidan and chitosan were also used to create fucospheres for the treatment of dermal burns in rabbits. As in the previous study, the size of the fucospheres obtained by polyion complexation increased with the increase in the polymer concentration. Wounds that were treated with fucospheres, were characterized by the highest epithelial thickness. The most relevant difference was observed on day 7th, when the value of epithelial thickness was 193, 121, 118, and 111 µm, for fucospheres, fucoidan solution, chitosan microspheres and untreated group, respectively. Similarly, epithelial lengths increased with time, and on the 14th day of therapy the highest values were observed in the group treated with fucospheres (2733, 2086, 2316, and 1950 µm, as above). The fastest skin regeneration after burns was noted in the group treated with fucospheres due to the effect of fucoidan on the migration of fibroblasts, release of growth hormones and cytokines involved in re-epithelization. In addition, based on this study, it was concluded that fucoidan and chitosan have a synergistic effect on the treatment of skin burns [48].

Li et al. designed poly(alkylcyanoacrylate) (PACA) microcapsules with fucoidan layer on their surface. The use of fucoidan was due to its ability to detect thrombosis by binding to P-selectin. The microparticles produced by emulsion–evaporation polymerization process were filled with contrast agent (perfluorooctylbromide—PFOB) used in various imaging diagnostics techniques (ultrasonography, magnetic resonance imaging). The safety of microparticles application was determined by MTT test using 3T3 mouse fibroblast cells. Even at concentration of 5 g/L, there was no difference in cell viability when using microcapsules with and without fucoidan. Additionally, two in vitro studies were performed to evaluate their binding capacity to P-selectin. Both in the static binding on human activated platelets and in the flow chamber assay, microparticles with fucoidan showed significant adhesive properties. Moreover, designed microcapsules were administered intravenously to healthy Wistar rats and rats with abdominal aortic aneurysm. The strongest fluorescence was observed in the animals treated with microcapsules with fucoidan but only in the affected area. Weaker bonds of both types of microparticles were noted in healthy blood vessels. It was concluded that fucoidan microcapsules seem to be valuable targeting carrier candidate for drug substances or contrast agents for the treatment or imaging of diseases that are accompanied by overexpression of P-selectin [71].

Fucoidan microparticles are also examined as potential drug carriers in the cancer treatment. In 2017, Wang et al. used the layer-by-layer self-assembly technique to develop microparticles with DOX. After coating the core made of calcium carbonate with PLO and fucoidan, they were loaded with DOX at a high, nearly 70% encapsulation efficiency. Designed microparticles were characterized by biocompatibility—they did not affect the survival of C2C12 mouse myoblasts (at concentration of 400 µg/mL cell viability was about 90%). In addition, the hemolysis rate at 200 µg/mL was lower than 3%. A study conducted at pH 7.4 showed prolonged DOX release, and antitumor tests on breast cancer cells (MCF-7) confirmed its effective release and ability to inhibit cells more than free DOX [72].

Fucoidan microparticles are also tested as antibiotics carriers. Sezer et al. compared the properties of fucospheres and chitosan microspheres, obtained by polyion complexation and precipitation methods, respectively. Ofloxacin (OFL) encapsulation efficiency (EE) was greater in fucospheres than chitosan microparticles (EE values 63.9%–94.8% and 48.8%–89.2%, respectively). The use of fucoidan caused a slower OFL release, consistent with the Higuchi kinetics model [73]. Recently, Cunha et al. conducted studies on the use of fucoidan-based microparticles in the treatment of tuberculosis. This is the result of fucoidan ability to recognize macrophages in the alveoli, which allows the delivery of drugs to the affected areas and leads to an increase in the effectiveness of the therapy. The microparticles containing isoniazid (INH) or rifabutin (RFB) produced by the spray-drying with high yield (75%–85%) were characterized by the appropriate aerodynamic properties measured using the Andersen cascade impactor (ACI). The mass median aerodynamic diameter of particles with isoniazid was about 3.78 µm, and with rifampicin 1.99 µm. The fraction of fine particles tested with the dry powder inhaler type RS01, showing the most effective penetration to the lungs, was 39% and 55%, respectively. Due to the wrinkled surface of the microparticles and the good solubility of fucoidan, the total release of both drugs occurred in a short time (about 15 minutes), regardless of the medium pH and the solubility of the free drug. It is worth noting that MTT tests proved that microencapsulation reduces the toxicity of loaded RFB, both in relation to human alveolar epithelium (A549) and human monocytic (THP-1) cells. The viability of A549 and THP-1 cells after 24 h incubation with RFB at a concentration of 0.05 mg/mL was 50% and 49%, respectively. In turn, the use of the microparticles with RFB increased cells viability after 24 h incubation even up to 90% (A549) and to around 70% (THP-1). However, unloaded fucoidan-based microparticles, free fucoidan, and free INH did not show cytotoxicity [43]. Very similar results were noted with reference to fucoidan-based microparticles being a combination of INF and RFB. The spray drying technique allowed to obtain product with a yield of over 80%. By using the RS01 inhaler in the ACI, it was found that product exhibited proper aerodynamic properties. In studies in vitro it was reported that microparticles did not affect the viability of A549 cells and their viability was classified above 70%, regardless the incubation time. THP-1 cells showed greater sensitivity and at concentration 1.0 mg/mL after 24 h of exposure, the viability decreased to 65%. Viability of A549 and THP-1 cells after 3 h incubation with free RFB (0.05 mg/mL) was 68% and 56%, whereas with microparticles (1.0 mg/mL) containing INH and RFB at mass ratio 1:0.5, 84% and 77%, respectively. It is worth noting that the significant uptake of microparticles by macrophage-like cells (up to 87%) along with their simultaneous stimulation for the production of cytokines involved in the fight against pathogen was associated with the presence of fucoidan [74].

### 4.4. Semi-Solid Formulations

Hydrogels are three-dimensional formulations made of hydrophilic polymers with appropriate structure and properties. They are widely used in biomedicine, for example as wound dressings, implants, drug delivery systems, or tissue regeneration materials [75].

Unique properties, such as anticoagulant and anti-inflammatory activity make fucoidan a useful component in the formation of hydrogels. Sezer et al. proposed a hydrogel obtained by combining fucoidan with an oppositely charged chitosan. An important feature of hydrogels is the ability to absorb exudate and to provide adequate moisture. It turns out that formulations with fucoidan addition were characterized by greater possibility of water absorption and higher swelling ratio than pure chitosan gels. Electrostatic interactions between polymers significantly affected the hardness of the gels, which increased with the increase of fucoidan concentration. Similarly to the parameters discussed above, formulations containing the highest concentrations of polymers (0.75% fucoidan and 2% chitosan) showed the greatest cohesiveness and adhesion values. Stronger mucoadhesive properties were also associated with the content of fucoidan. Hydrogels containing only chitosan and chitosan with the addition of 0.25% or 0.75% fucoidan showed the following values of work of adhesion 23, 62, and 142 µJ/cm^2^, respectively. The effectiveness of wound healing using the designed hydrogels has also been evaluated in vivo. In a study conducted on New Zealand rabbits statistically significantly higher values of the length and thickness of the epithelium were observed in wounds treated with fucoidan–chitosan gel. In addition, the number of rete pegs, responsible for fixing the epidermis in the dermis and organized nuclear regions (NOR), which confirmed cell proliferation, were also the highest in this group. Complete cure within 21 days was observed only with the use of a complex hydrogel, affirming the synergistic effect of fucoidan and chitosan on the healing process [76].

In turn, Murakami et al. proposed hydrogels made of chitosan/chitin, fucoidan and alginate. Hydrogels obtained by crosslinking with ethylene glycol diglycidyl ether were safe and showed no cytotoxicity to human dermal fibroblast (DFC) and dermal microvascular endothelial (DMVEC) cells. In comparison to the commercially available product (Kaltostat—calcium alginate fiber), they were characterized by better—gradually increasing exudate absorption capacity, without maceration for 18 h. In vivo studies in rats with mitomycin C-induced, impaired wound healing confirmed the positive effects of fucoidan—its ability to interact with growth factors (FGF-1 and FGF-2) and with cytokines involved in the reconstruction of the epidermis and angiogenesis processes. The stimulation of the granulation and capillary formation observed after 7 days of treatment with hydrogel contributed to the best results after 18 days of application, including effective division of epidermal stem cells located in the intact adjacent epidermis and the strongest wound closure. Interestingly, the effect of the hydrogel on the course of wound healing without the factor inhibiting cell proliferation (mitomycin C) was insignificant [77]. Hydrosheets made of the same polymers (fucoidan, chitin/chitosan, and alginate) were compared with two different hydrogel dressings (Kaltostat and DuoACTIVE). The obtained results indicated an advantage in the treatment of mitomycin C-impaired wounds with the proposed dressings than with the currently available products or plastic wrap used as a control (in the study group within 14 days there was a reconstruction of the epidermis with significantly higher wound closure rate). It should be added that the use of all these dressings had no significant effect on the wounds which were not treated with mitomycin C [78].

As fucoidan is characterized by the ability to interact with growth factors and possesses lower than heparin anticoagulant activity, it might be utilized to create drug carrier for tissue regeneration. The main limitation in designed carriers for protein compounds, is their short half-life in vivo, resulting from sensitivity to temperature and enzymes. Nakamura et al. formed a micro complex-hydrogel composed of chitosan and fucoidan loaded with fibroblast growth factor-2 (FGF-2). The gradual release of FGF-2 along with progressive degradation of the hydrogel contributed to statistically significantly stronger neovascularization in the test group compared to mice injected with growth factor alone or hydrogel placebo [79]. In turn, vascular endothelial growth factor (VEGF) and the influence of fucoidan molecular weight on its activity in micro- and macroporous 3D scaffolds were evaluated by Purnama et al. Hydrogels obtained by crosslinking with sodium trimetaphosphate (STMP) pullulan and DXT were control group, and those with fucoidan (with low, medium, and high molecular weight—LMWF/MMWF/HMWF) addition were test groups. It was shown that significant decrease in the release rate of VEGF compared to the control, regardless of the pore size in the hydrogel, guaranteed the addition of fucoidan with medium molecular weight (39 kDa). A positive aspect is also the significant increase in the number of human endothelial progenitor cells and their extent of proliferation. The synergism of MMWF and VEGF was confirmed by observations after subcutaneous injection of hydrogels to C57/BL6J mice. Both the neovessel area and density in the group treated with hydrogels obtained from MMWF and VEGF were statistically higher compared with control group or group treated scaffolds with single substance [80].

In order to obtain hydrogel to ocular applications made of poly(2-hydroxyethyl methacrylate) (pHEMA), fucoidan and methacrylic acid (MAA), Lee at al. used ethylene glycol dimethacrylate (EGDMA) as crosslinking agent. Developed hydrogels were tested in terms of water absorption capacity, adsorption of proteins and antibacterial properties. It was noted that water content of hydrogels and adsorption of proteins (especially lysozyme) did not depend on the concentration of fucoidan and increased only with MAA increase. The predicted antibacterial activity of fucoidan with regard to *S. aureus* and *E. coli* turned out to be infinitesimal, and its potentiation was caused by the addition of MAA [81].

## 5. Conclusions

Fucoidan is the important ingredient in various pharmaceutical formulations. It can be used either as a substance with a specific therapeutic effect or as an excipient, allowing to obtain a drug form with appropriate properties. However, utilization of fucoidan in the pharmaceutical industry involves many challenges. Difficulties appear already at the acquisition stage. Differences in the growth conditions of algae, pollution of the marine environment, as well as a wide range of species that are the source of fucoidan contribute to the heterogeneity of its properties. This, in turn, leads to the necessity of matching extraction and purification methods to allow obtaining the raw material with desired characteristics [82,83,84]. Fucoidan features are also influenced by sugar composition and sulfate content, and by differential molecular weight (10–2000 kDa). Other disadvantage is lack of gelation ability, important feature in designing drug dosage forms. This limitation can be overcome by fucoidan combining with polymers (chitosan and its derivatives) or other compounds that provide a positive charge (protamine, polyethyleneimine, polyallyamine hydrochloride, poly(isobutylcyanoacrylate), poly(lactide-co-glycolide), poly-l-orithine, and hexadecylamine, poly(alkylcyanoacrylate)). The broad spectrum of biological activity of this polysaccharide per se contributes to the fact that application of designed drug carriers, like nanoparticles, liposomes, microparticles or semi-solid forms is very differential and using of fucoidan not only provides protection of the loaded active substance, but can also increase its potency and improve the effectiveness of the treatment. Due to diversified properties and potential broad area of application, the interest in fucoidan utilization in the biomedicine and pharmaceutical industry will be probably extended.

## Figures and Tables

**Table 1:** Effect of fucoidan concentration on the pH value, viscosity and color of its aqueous solutions.

Concentration of Fucoidan [% *w/w*]	pH Value	Viscosity^1^	Color
1	5.23	-	yellowish
2	5.05	3.5	slightly yellow
3	4.96	4.2	yellow
5	4.72	7.2	dark yellow
10	4.61	18.8	brownish
20	4.47	100.8	brown
30	4.45	507.0	dark brown

^1^ Viscosity of prepared aqueous solutions was determined using Viscotester 6 Plus ThermoHaake. (Thermo Scientific, Karlsruhe, Germany) equipped with a rotor TL 5 at 25 °C ± 1 °C.

**Table 2:** Characteristic of selected fucoidan-based nanoparticles.

Fucoidan (Source/Modification/Molecular Weight)	Copolymer/Positive Charge Donor	Drug	Method of Obtaining	Application	Route of Administration	Ref.
Acetylated fucoidan (*Fucus vesiculosus*)	-	Doxorubicin	Self-assembly and dialysis	Anticancer therapy and immunotherapy	NA^1^	51
Fucoidan (*Laminaria japonica*, 80 kDa)	Protamine	Doxorubicin	Self-assembly	Anticancer therapy	Intravenous	52
Fucoidan (*Fucus vesiculosus*)	Polyethyleneimine	Doxorubicin	Polyelectrolyte complexation method	Anticancer therapy	Intravenous	53
Fucoidan (*Fucus vesiculosus*)	Gold nanoparticles	Doxorubicin	Electrostatic physisorption	Anticancer therapy	Ocular	42
Fucoidan (*Fucus vesiculosus*)	Polyallylamine hydrochloride	Copper sulfide	Layer-by-layer	Anticancer therapy	Intratumoral	54
Fucoidan (200–400 kDa)	Polyallyamine hydrochloride	Methotrexate	Self-assembly	Anticancer therapy	NA^1^	55
Fucoidan (*Fucus vesiculosus*)	Chitosan	-	Coacervation	Thrombolytic therapy	Oral	56
Fucoidan (*Fucus vesiculosus*, 50–190 kDa)	Chitosan	Methotrexate	Self-assembly	Skin inflammation	Topical (ear skin)	57
Fucoidan	Chitosan	Curcumin	Self-assembly	Anticancer therapy	Oral	58
Fucoidan (*Fucus vesiculosus*)	O-carboxymethyl chitosan	Curcumin	Ionotropic crosslinking	Penetration enhancer	Oral	59
Thiolated fucoidan (THL-fucoidan)	Arginine-modified chitosan	Dextran/rhodamine/curcumin	Self-assembly	NA^1^	Oral	60
Fucoidan (*Fucus vesiculosus*)	Chitosan	Gentamicin	Self-assembly	Pulmonary diseases	Pulmonary	41
Fucoidan (*Fucus vesiculosus*)	Chitosan	Gentamicin	Ionotropic crosslinking	Pulmonary diseases	Pulmonary	50
Fucoidan (*Fucus vesiculosus*)	Chitosan	Silver nitrate	Self-assembly	Antibacterial and anticancer therapy	NA^1^	61
Fucoidan (20–200 kDa)	TPP crosslinked chitosan	Ciprofloxacin	Self-assembly	Infections of *Salmonella*	NA^1^	40
Fucoidan (*Fucus vesiculosus*, 57.26 kDa)	Chitosan	Poly-l-lysine	Layer-by-layer	Antibacterial therapy	NA^1^	62
Fucoidan (*Fucus vesiculosus*, 5–50 kDa)	Trimethyl chitosan	Insulin	Self-assembly	Diabetes	Oral	63
Fucoidan (*Fucus vesiculosus*, 80 kDa)	Chitosan	Basic fibroblast growth factor	Ionotropic crosslinking	Neurite extension	Nerve tissue	44
Fucoidan (104 kDa)	Isobutylcyanoacrylate	Recombinant tissue plasminogen activator	Redox radical emulsion polymerization	Thrombolytic therapy	Retro-orbital (C57BL/6 mice)	64
Fucoidan (*Sargassum cymosum*)	Isobutylcyanoacrylate	-	Anionic emulsion polymerization and redox radical emulsion polymerization	Immunotherapy	NA^1^	65
Fucoidan	Poly(lactide-co-glycolide) and poly-l-ornitine (core-shell)	-	Layer-by-layer	Anticancer therapy	NA^1^	66
Fucoidan (*Fucus vesiculosus*, 20–200 kDa)	-	Cisplatin	Self-assembly	Anticancer therapy and immunotherapy	Colonic drug delivery system	67
Fucoidan (*Spatoglossum schrőederi*, 21 kDa)	Hexadecylamine	-	Self-assembly	Anticancer therapy	NA^1^	68

^1^NA—No data available.

**Table 3:** Characteristic of selected fucoidan-based microparticles.

Fucoidan (Source/Molecular Weight)	Copolymer/Positive Charge Donor	Drug	Method of Obtaining	Application	Route of Administration	Ref.
Fucoidan (*Fucus vesiculosus*, 80 kDa)	Chitosan	Bovine serum albumin	Ionotropic cross-linking	Peptide and protein delivery	NA ^1^	45
Fucoidan (*Fucus vesiculosus*, 80 kDa)	Chitosan	-	Polyion complexation	Treatment of dermal burns	Topical	48
Fucoidan	Poly(alkylcyanoacrylate)and dextran	Perfluorooctylbromide	Emulsion-evaporation polymerization	Targeting carrier	Intravenous	71
Fucoidan (200-400 kDa)	Poly-l-ornithine (shell); calcium carbonate (core)	Doxorubicin	Layer-by-layer self-assembly	Anticancer therapy	NA ^1^	72
Fucoidan (*Fucus vesiculosus,* 80 kDa)	Chitosan	Ofloxacin	Polyion complexation	Antibiotics carriers	NA ^1^	73
Fucoidan (*Laminaria japonica*, 598.4 Da–0.598 kDa)	-	Isoniazid or rifabutin	Spray-drying	Tuberculosis therapy	Pulmonary	43
Fucoidan (*Laminaria japonica*)	-	Isoniazid and rifabutin	Spray-drying	Tuberculosis therapy	Pulmonary	74

^1^ No data available.
